# The use of antiepileptic drugs and their effects in pregnancy

**DOI:** 10.55730/1300-0144.5950

**Published:** 2024-11-26

**Authors:** Pınar ÖZTÜRK, Ali Turhan ÇAĞLAR

**Affiliations:** 1 Department of Neurology, Ankara Yenimahalle Education and Research Hospital, Ankara, Turkiye; 2 Department of Gynecology, Ankara Etlik City Hospital, Ankara, Turkiye

**Keywords:** Epilepsy, pregnancy, seizure

## Abstract

**Background/aim:**

Epilepsy is one of the most common chronic neurological diseases. It can affect patients throughout their entire lives, including the reproductive years. It is estimated that epileptic women account for three to five births per 1000 births. Although the use of antiepileptic drugs (AEDs) during pregnancy has been associated with major congenital malformations in the fetus, many patients cannot discontinue the drugs before pregnancy due to the risk of seizures that may harm the mother as well as the child. In this study, our aim was to examine the follow-up findings of pregnant women who were epileptic and using AEDs during pregnancy and delivery, and to contribute to the literature on this subject, for which studies are still ongoing.

**Materials and methods:**

Sixty epileptic pregnant women and 60 healthy pregnant women were included in the study. These women gave birth at the University of Health Sciences Zekai Tahir Burak Women’s Health Application and Research Center. The women in the case group had applied to the neurology outpatient clinic between April 1, 2018, and April 1, 2019; were previously diagnosed with epilepsy by a neurologist; had started using AEDs before pregnancy; and had used AEDs in the first trimester.

**Results:**

While the number of pregnancies per woman in the case group (1.75 ± 0.77) was significantly lower than that in the control group (2.25 ± 1.17), the rate of cesarean section deliveries and neonatal intensive care needs were found to be statistically significantly higher in the case group (68.3% and 20.0%, respectively) than in the control group (43.3% and 5.0%, respectively) (p < 0.05). There was no statistically significant difference between the groups in terms of birth week or birth weight.

**Conclusion:**

Epileptic pregnancies have unique risks, differing from normal pregnancies. It is important that pregnant women with epilepsy be followed closely from the pregnancy planning period to delivery in cooperation with the physician.

## 1. Introduction

Epilepsy is one of the most common chronic neurological diseases and it is seen in the general population with a frequency of 4–10 per 1000 [1]. Epilepsy can affect patients throughout their entire lives, including the reproductive years. It is estimated that epileptic women account for 3–5 births per 1000 births [2]. Although the use of antiepileptic drugs (AEDs) during pregnancy has been associated with major congenital malformations in the fetus, many patients cannot discontinue the drugs before pregnancy due to the risk of seizures that may harm the mother as well as the child [3]. Therefore, children of women with epilepsy have a two- to threefold increased risk of congenital malformations compared to the general population [4].

Congenital malformations in the infants of pregnant women with epilepsy are categorized as major and minor. Malformations that can cause death or serious loss of function are referred to as major congenital malformations [5]. The gestational period in which major congenital malformations are most common is the first trimester, when organogenesis occurs [6]. The most common major congenital malformations are cardiac defects, urogenital malformations, craniofacial defects, and skeletal anomalies, as in the general population [7]. While malformations are seen at a rate of 1.6% to 3.2% in the general population, they are seen at a rate of 2.3% to 7.8% in the infants of women receiving AED monotherapy and at a rate of 6.5% to 16.8% in cases of AED polytherapy [5]. Malformations that do not cause death or serious loss of function are considered as minor congenital malformations. These malformations include dysmorphic face, low birth weight, hypoplasia of the nail distal phalanx, and decreased height and head circumference [8]. These are seen at rates 2–2.5 times higher than those observed in the general population [9].

The most important goals in epileptic pregnancy follow-up are to maintain the drugs at the lowest dose that provides seizure control and to use a single drug if possible. Preconception planning and drug dose adjustment are very important for a healthy pregnancy and healthy birth. In this study, our aim was to examine the follow-up findings of pregnant women who were epileptic and using AEDs during pregnancy and delivery, and to contribute to the literature on this subject, for which studies are still ongoing.

## 2. Materials and methods

Sixty epileptic pregnant women were included in this study. These women gave birth at the University of Health Sciences Zekai Tahir Burak Women’s Health Application and Research Center; applied to the neurology outpatient clinic between April 1, 2018, and April 1, 2019; were previously diagnosed with epilepsy by a neurologist; and had started using AEDs before pregnancy and continued using AEDs in the first trimester.

The study was retrospective and the data analyzed in the study were acquired from neurology and gynecology and obstetrics outpatient clinic examinations and file information. The analyzed parameters included the physical and neurological examination findings of the patients, their ages and durations of epilepsy, the AEDs and doses they were taking, the number of seizures during pregnancy, the number of pregnancies, the types and outcomes of deliveries, EEG findings, malformations occurring in the infants, and the birth measurements of the infants.

As the control group, 60 pregnant women were included. These women gave birth at the University of Health Sciences Zekai Tahir Burak Women’s Health Application and Research Center and were randomly selected for retrospective analysis based on file numbers ending with the number 9.

The University of Health Sciences Zekai Tahir Burak Women’s Health Application and Research Center’s Medical Specialization Education Board approved this study on December 28, 2018 (Decision No. 26).

### 2.1. Statistical analysis

Continuous variables were expressed as mean ± standard deviation and median (minimum–maximum), and categorical data were expressed as numbers and percentages. In the intergroup analysis of continuous variables, normality analysis was performed with the Kolmogorov–Smirnov goodness-of-fit test. While t-tests were used in the analysis of continuous variables between two groups with normal distribution, the Mann–Whitney U test was used for those without normal distribution. Comparisons between three groups of variables that did not comply with normal distribution were performed with the Kruskal–Wallis test. The chi-square test (with Fisher’s exact test and linear-by-linear association subtests when necessary) was used to compare categorical data. Analysis was performed with IBM SPSS Statistics 24.0 (IBM Corp., Armonk, NY, USA). Statistical significance was accepted at p < 0.05.

## 3. Results

There was no significant difference between the age distributions of the case and control groups (p < 0.05). While the number of pregnancies per woman in the case group (1.75 ± 0.77) was significantly lower than that in the control group (2.25 ± 1.17), the rate of cesarean section delivery and neonatal intensive care needs were found to be statistically significantly higher in the case group (68.3% and 20.0%, respectively) than in the control group (43.3% and 5.0%, respectively) (p < 0.05). There was no statistically significant difference between the groups in terms of birth week or birth weight (Table 1).

None of the pregnant women with epilepsy in this study had any additional comorbidities.

When a comparison was made according to the types of AEDs used by women in the case group, the median duration of drug use for lamotrigine was 8 years, ranging from 1 to 17 years; for levetiracetam, the median was 6 years, ranging from 1 to 27 years; for valproic acid, the median was 12 years, ranging from 3 to 15 years; for carbamazepine, the median was 7 years, ranging from 1 to 20 years; and for oxcarbazepine, the median was 8.5 years, ranging from 1 to 27 years. There was no significant difference between the durations of drug use (p > 0.05).

For lamotrigine users, 27.3% of births occurred before week 37, while 72.7% occurred between weeks 37 and 42. For levetiracetam users, 11.1% of births occurred before week 37, while 88.9% occurred between weeks 37 and 42. For valproic acid users, 33.3% of births occurred before week 37, while 66.7% occurred between weeks 37 and 42. For carbamazepine users, 6.7% of births occurred before week 37, while 93.3% occurred between weeks 37 and 42. For oxcarbazepine users, all (100%) births occurred between weeks 37 and 42. There was no significant difference between the birth weeks of these infants (p > 0.05).

Infants with low birth weights (<2500 g) were born to two (18.8%) of 11 patients treated with lamotrigine and one (33.3%) of three patients treated with valproic acid, and it was found that these rates were statistically significantly different compared to other drug groups (p < 0.05).

Among lamotrigine users, 18.8% of infants weighed less than 2500 g, while 81.8% weighed 2500 g or more. Among levetiracetam users, all (100%) infants weighed 2500 g or more. Among valproic acid users, 33.3% of infants weighed less than 2500 g, while 66.7% weighed 2500 g or more. Among carbamazepine users, all (100%) infants weighed 2500 g or more. Among oxcarbazepine users, all (100%) infants weighed 2500 g or more. No significant difference was detected between the birth types of the infants (i.e. cesarean section versus vaginal delivery).

While generalized onset seizures were observed in patients treated with lamotrigine, levetiracetam, and valproic acid (90.9%, 94.4%, and 100.0%, respectively), focal onset seizures were observed in those treated with carbamazepine and oxcarbazepine (73.3% and 100%, respectively). While eight (72.7%) of 11 patients treated with polytherapy had generalized onset seizures, three (27.3%) had focal onset seizures (p < 0.001).

While ≥2 seizures were observed during pregnancy in 18.2% of the patients treated with lamotrigine, this rate was 33.3% with levetiracetam, 26.7% with carbamazepine, and 45.5% with polytherapy. However, ≥2 seizures were not detected in any patients treated with valproic acid or oxcarbazepine (p > 0.05). The frequency of the use of polytherapy increased as the number of seizures increased.

No statistically significant difference was detected between types of AEDs in terms of abnormal EEG findings or neonatal intensive care needs (p > 0.05) (Table 2).

The average doses of drugs used by the patients were 150 mg for lamotrigine, 1000 mg for levetiracetam, 500 mg for valproic acid, 400 mg for carbamazepine, and 450 mg for oxcarbazepine (Table 3). The drug combination and doses for polytherapy use are provided in Table 4.

Congenital malformations were observed in three of the 60 infants born to epileptic pregnant women. The first of these patients was 30 years old and was using levetiracetam at 1000 mg/day. She gave birth by cesarean section at week 35. The infant’s birth weight was 3850 g and patent foramen ovale was observed. The second patient was 23 years old and was using carbamazepine at 400 mg/day. She gave birth by cesarean section at week 40. The infant’s birth weight was 3250 g and hydronephrosis was observed. The third patient was 29 years old and was using lamotrigine at 200 mg/day. She had a normal vaginal delivery at week 36. The infant’s birth weight was 2950 g and an undescended testicle was observed. The reasons for these malformations may be both the use of drugs during the organogenesis period and the effect of epileptic seizures during pregnancy.

## 4. Discussion

More than 90% of pregnancies of epileptic women have healthy outcomes. However, an increase in maternal and fetal complications is observed in epileptic pregnant women [10]. AED use and seizures during pregnancy increase these risks. Increased risks of abortion, low birth weight, developmental delay in mental motor functions, and congenital malformation in infants of pregnant women using AEDs have been reported [11]. Increased estrogen and progesterone levels during pregnancy may also affect the occurrence of epileptic seizures [12].

The general opinion is that the risk of malformation increases by 2- to 3-fold in infants of epileptic pregnant women compared to the general population [13,14]. In some studies, while the malformation rate was found to vary between 2% and 3% in the general population, rates of 1.25%–11.5% were reported for AED users [15].

In epileptic pregnancies, normal vaginal delivery is possible if the patient is obstetrically suitable. The fact that the pregnant woman has epilepsy alone is not an indication for cesarean delivery and the decision is made according to the patient’s general condition, the frequency of seizures in the last trimester, and the factors triggering the seizures. Although vaginal delivery is recommended for pregnant women with epilepsy, it is suggested that the unpredictable stress of birth, seizures due to insomnia, and the uncertainty of possible complications during delivery may increase the rate of cesarean section deliveries in patients with epilepsy [16]. In our study, the cesarean section rate and neonatal intensive care needs were found to be statistically significantly higher in the case group (68.3% and 20.0%, respectively) than in the control group (43.3% and 5.0%, respectively) (p < 0.05). When the 41 cases of cesarean section were reviewed, it was seen that 32 of them were performed for obstetric reasons and nine were performed due to neurological indications. There was no statistically significant difference between the groups in terms of birth week or birth weight (Table 1). Even in the absence of malformations, the need for neonatal intensive care was found to be increased in the infants of epileptic women compared to the control group. In a similar study conducted by Karademir et al., cesarean section delivery rates were found to be as high as 66.2% in epileptic pregnant women [17].

In our study, when the drugs used by the patients with epilepsy were compared, no significant difference was detected between birth weeks, birth weights, or delivery types of the infants according to the AEDs used by the mothers (p < 0.05). However, two (18.8%) of 11 patients treated with lamotrigine and one (33.3%) of three patients treated with valproic acid gave birth to infants with low birth weights (<2500 g), and these rates constituted a statistically significant difference compared to the other drug groups (p < 0.05). In a large-scale study including 4342 patients, rates of major malformation were reported as 9.3% for valproic acid, 5.5% for phenobarbital, 4.2% for topiramate, 3% for carbamazepine, 2.9% for diphenylhydantoin, 2.4% for levetiracetam, and 2% for lamotrigine among the infants of pregnant women using AEDs [18]. The absence of malformation in pregnant women using valproic acid in Türkiye may be due to the low overall number of patients using valproic acid. The risk of congenital malformations in epileptic pregnant women increases in proportion to the number of AEDs used and with increasing AED doses. During pregnancy, the lowest possible dose and minimum number of AEDs are recommended [16,19,20]. However, the mothers of the three infants observed to have malformations in the present study were monotherapy patients and their drug doses were within the recommended safe dose ranges.

Lacosamide is a new drug and not many studies have been conducted to date on its use by epileptic pregnant women. One of our patients was using oxcarbazepine at 1200 mg/day, levetiracetam at 3000 mg/day, and lacosamide at 250 mg/day. She had a normal vaginal delivery at week 37 and gave birth to a healthy male infant weighing 3130 g.

There are some limitations of this study to be noted. This study had a small sample size and was conducted at a single center. Since the hospital in which the study was conducted was specifically an obstetrics and gynecology hospital, prepregnancy epilepsy data were not available.

The strengths of this study are the direct access to all patients’ delivery information and the fact that the study was conducted at a site experienced in high-risk pregnancy follow-up. In addition, it is expected that this analysis of 60 epileptic pregnant women and the provision of insights in terms of the effects of new AEDs during pregnancy will contribute to the literature on drug effects in epileptic pregnant women.

## Figures and Tables

**Table 1:** Comparison of some descriptive and clinical parameters of the patient groups.

	Case group (n = 60)	Control group (n = 60)	p

**Age (years) (Mean ± SD)**	28.83 ± 6.17	27.76 ± 4.86	0.295*
**(Median, min-max)**	28.5 (18–41)	28 (18–38)	

**Number of pregnancies (Mean ± SD)**	1.75 ± 0.77	2.25 ± 1.17	**0.026** **
**(Median, min-max)**	2 (1–4)	2 (1–5)	

**Birth week (n, %)**			1.000***^a^
**< Week 37**	7 (11.7%)	8 (13.3%)	
**Week 37–42**	53 (88.3%)	52 (86.7%)	
**(Median, min-max)**	38 (33–41)	39 (34–41)	

**Birth weight (n, %)**			1.000***^a^
**<2500 g**	2 (3.3%)	3 (5.0%)	
**2500 g and above**	58 (96.7%)	57 (95.0%)	
**(Median, min-max)**	3200 (1850–4480)	3300 (2440–4500)	

**Type of birth (n, %)**			**0.006** ***
**Vaginal**	19 (31.7%)	34 (56.7%)	
**Cesarean**	**41 (68.3%)**	**26 (43.3%)**	

**Neonatal intensive care need**			**0.025** *** ^a^
**None**	48 (80.0%)	57 (95.0%)	
**Yes**	**12 (20.0%)**	**3 (5.0%)**	

**Total**	60 (100.0%)	60 (100.0%)	

* t test,

** Mann–Whitney U test,

*** chi-square test (Fisher’s exact test ^a^).

**Table 2:** Comparison of some clinical parameters of the case group according to drug use status.

	Lamotrigine (n = 11)	Levetiracetam (n = 18)	Valproic acid (n = 3)	Carbamazepine (n = 15)	Oxcarbazepine (n = 18)	Polytherapy (n = 11)	p

**Duration of drug use -years (Median, min-max)**	8 (1–17)	6 (1–27)	12 (3–15)	7 (1–20)	8.5 (1–27)	8 (2–20)	0.936*

**Birth week (n, %)**							0.546**
**<Week 37**	3 (27.3%)	2 (11.1%)	1 (33.3%)	1 (6.7%)	0 (0.0%)	1 (9.1%)	
**Week 37–42**	8 (72.7%)	16 (88.9%)	2 (66.7%)	14 (93.3%)	2 (100.0%)	10 (90.9%)	

**Birth weight (n, %)**							**0.042** **
**<2500 g**	**2 (18.8%)**	0 (0.0%)	**1 (33.3%)**	0 (0.0%)	0 (0.0%)	0 (0.0%)	
**2500 g and above**	8 (81.8%)	18 (100.0%)	2 (66.7%)	15 (100.0%)	2 (100.0%)	11 (100.0%)	

**Type of birth (n, %)**							0.112**
**Vaginal**	5 (45.5%)	2 (11.1%)	1 (33.3%)	5 (33.3%)	2 (100.0%)	4 (36.4%)	
**Cesarean**	6 (54.5%)	16 (88.9%)	2 (66.7%)	10 (66.7%)	0 (0.0%)	7 (63.6%)	

**Seizure type (n, %)**							**<0.001** **
**Generalized**	**10 (90.9%)**	**17 (94.4%)**	**3 (100.0%)**	4 (26.7%)	0 (0.0%)	8 (72.7%)	
**Focal**	1 (9.1%)	1 (5.6%)	0 (0.0%)	**11 (73.3%)**	**2 (100.0%)**	3 (27.3%)	

**Number of seizures had (n, %)**							0.541**
**No seizure**	7 (63.6%)	10 (55.6%)	2 (66.7%)	10 (66.7%)	2 (100.0%)	3 (27.3%)	
**1 seizure**	2 (18.2%)	1 (11.1%)	1 (33.3%)	1 (6.7%)	0 (0.0%)	3 (27.3%)	
**≥ 2 seizures**	2 (18.2%)	6 (33.3%)	0 (0.0%)	4 (26.7%)	0 (0.0%)	**5 (45.5%)**	

**EEG (n, %)**							0.559**
**Pathological**	5 (45.5%)	7 (38.9%)	0 (0.0%)	5 (33.3%)	1 (50.0%)	3 (27.3%)	
**Normal**	6 (54.5%)	11 (61.1%)	3 (100.0%)	10 (66.7%)	1 (50.0%)	8 (72.7%)	

**Neonatal IC need**							0.663**
**None**	9 (81.8%)	15 (83.3%)	2 (66.7%)	13 (86.7%)	2 (100.0%)	7 (63.6%)	
**Yes**	2 (18.2%)	3 (16.7%)	1 (33.3%)	2 (13.3%)	0 (0.0%)	4 (36.4%)	

**Total**	11 (100.0%)	18 (100.0%)	3 (100.0%)	15 (100.0%)	2 (100.0%)	11 (%100.0)	

* Kruskal–Wallis test,

** chi-square test.

**Table 3:** Median doses of drugs used for monotherapy in the case group.

	Lamotrigine	Levetiracetam	Valproic acid	Carbamazepine	Oxcarbazepine
**Drug dose [median (min-max)]**	150 (50–200)	1000 (750–2250)	500 (500–1000)	400 (300–800)	450 (300–600)

**Table 4:** Drug combinations and doses used for polytherapy in the case group.

	Drug combination administered	Drug dose (mg)
**1**	**Levetiracetam + ccarbamazepine**	1500 + 400
**2**	**Oxcarbazepine + levetiracetam + lacosamide**	1200 +3000 + 250
**3**	**Lamotrigine + valproic acid**	100 + 500
**4**	**Lamotrigine + carbamazepine**	125 + 200
**5**	**Levetiracetam + carbamazepine**	2000 + 800
**6**	**Lamotrigine + levetiracetam**	100 + 2000
**7**	**Levetiracetam + oxcarbazepine**	1000 + 1200
**8**	**Lamotrigine + valproic acid**	350 + 500
**9**	**Lamotrigine + carbamazepine**	200 + 400
**10**	**Levetiracetam + carbamazepine**	1500 + 800
**11**	**Lamotrigine + carbamazepine**	300 + 1500
